# Inferring Meaningful Communities from Topology-Constrained Correlation Networks

**DOI:** 10.1371/journal.pone.0113438

**Published:** 2014-11-19

**Authors:** Jose Sergio Hleap, Christian Blouin

**Affiliations:** 1 Department of Biochemistry and Molecular Biology, Dalhouise University, Halifax, Nova Scotia, Canada; 2 Department of Computer Science, Dalhouise University, Halifax, Nova Scotia, Canada; Technical University Darmstadt, Germany

## Abstract

Community structure detection is an important tool in graph analysis. This can be done, among other ways, by solving for the partition set which optimizes the modularity scores 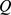. Here it is shown that topological constraints in correlation graphs induce over-fragmentation of community structures. A refinement step to this optimization based on Linear Discriminant Analysis (LDA) and a statistical test for significance is proposed. In structured simulation constrained by topology, this novel approach performs better than the optimization of modularity alone. This method was also tested with two empirical datasets: the Roll-Call voting in the 110th US Senate constrained by geographic adjacency, and a biological dataset of 135 protein structures constrained by inter-residue contacts. The former dataset showed sub-structures in the communities that revealed a regional bias in the votes which transcend party affiliations. This is an interesting pattern given that the 110th Legislature was assumed to be a highly polarized government. The 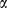-amylase catalytic domain dataset (biological dataset) was analyzed with and without topological constraints (inter-residue contacts). The results without topological constraints showed differences with the topology constrained one, but the LDA filtering did not change the outcome of the latter. This suggests that the LDA filtering is a robust way to solve the possible over-fragmentation when present, and that this method will not affect the results where there is no evidence of over-fragmentation.

## Introduction

Many problems in science can be abstracted as networks. For example, in biological sciences, protein structures can be abstracted as graphs of connected residues [1], metabolic networks can be created by connecting enzymes by their interactions in a given pathway [2], or food webs can be created by joining species with their trophic interactions [3]. Networks are common models for the Internet [4] and social networks [5]. Any kind of data that can be summarized into vertices (nodes) and connections (edges), can be abstracted as a graph. An special case of graphs can be constructed when one is interested in the correlation among variables. In this case, a correlation network can be constructed by assigning each variable to a vertex (or node), and the connections between are defined by the correlation. Since correlation is a measure of strength of relationship, the actual correlation value can be use as a weight in the edge, therefore representing such relationship. This graph abstraction is useful since allow us to analyze the relationships using the graph invariants. There are many such properties, but one of special interest here is the community structure which represents how the vertices are arranged in groups densely connected internally and sparsely connected externally [6].

Many networks have heterogeneous edge densities, which may imply a community structure. Communities are groups of nodes whose associations imply new insights in the understanding of a system [7]. A community can be loosely defined as groups of nodes that share more among themselves than to the rest of the graph. The most commonly used algorithm (and the one of focus in this paper) to detect communities in graphs is the modularity optimization proposed by Newman and Girvan [8]. In this algorithm, the modularity score 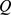 is optimized to obtain a partition scheme. Intuitively, 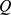 evaluates the excess of the number of edges inside a group against the expected connectivity of a randomly connected graph with similar properties. It can be calculated with: 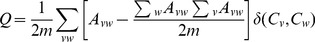
(1)where 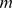 is the number of edges in the graph, 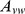 represents the weight of the edge between vertices 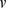, and 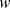, 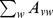 and 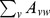 are the weighted degree of a vertex (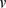 or 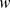), defined to be the sum of the edge weights of the adjacent edges for each vertex. 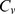 and 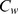 are communities to which the vectors 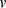 and 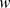 belong to, and the 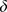 is a binary function where 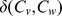 is 1 if 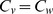 and 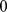 otherwise.

This approach has been applied to numerous problems [7], [9], [10]. Despite its wide use, exact algorithms for modularity optimization are computationally expensive. Some caveats also exist [7]: One example is the fact that high 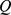 can be found in random graphs [11]. This issue might create either an over-fragmentation of the graph into smaller communities, or a failure to detect a small community which size is below a preset resolution limit [12]. Despite these caveats, modularity optimization (and in general community structure detection) is still an important tool in science if the confidence in the robustness of the solution can be assessed. Other methods to re-construct graphs and assess their structure exist, particularly dealing with high-dimensional data. Methods such as sparse graphical models [13] and LASSO-type problems [14] can be applied in graph reconstruction, and sometimes in community structure detection [15]. However, most of these methods rely on the assumption of independence of the variables [14] (or at least that the covariates are not highly correlated [16]), on the *a priori* determination of the number and size of the communities [15], and a full sparcity of the covariation among traits in the data. These kind of limitations makes these particular methods of limited in use in correlation networks, where the covariates are normally correlated, non-independent, and not completely sparse.

There is no guarantee that a community based on correlation is actually meaningful. It is posited here that asserting the statistical significance of a community enhances the odds that such structure provides insight. An application in protein structures exploring this with a Cholesky decomposition-based simulation have previously been shown [1]. After the membership vector is created by the optimization of 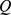, a pairwise permutation test is used to evaluate the statistical significance of each bipartition between modules. If the test fails, the two modules are merged and the membership vector is iteratively refined. In this work [1], the performance of community inference was shown to be high for simulated data.

Let us consider the case of correlation networks, where the edges are defined as the correlation between two nodes. These networks are important in biological sciences [1], [17]–[19] and economics [20], [21] since they constitute an intermediate between topology and the dynamics of the system [22]. Analyzing the community structures of these networks can help identify clusters of co-expressed genes causing a disease, or groups of stocks that are co-varying in the market. It is important to know whether such clustering partition has any significance. In some cases it is also appropriate to constraint a graph to a meaningful topology. For example: let's define a correlation network as a graph where two vertices are connected by an edge with a weight determined by the correlation of a pair of properties. It is also possible to further define a topologically-constrained correlation graph as a graph where an edge would exist only if the two incident vertices are connected by another meaningful property. The extra constraint in topology will create a sparser graph. Sparser graphs show an intrinsic level of modularity due to their topologies [23]. This is a problem if the modularity is inferred on the assumption that the community structure is dictated by correlation. It has also been shown that sparser graphs tend to cluster into more modules than predicted before [24]. Let's define this effect as over-fragmentation. In some cases the sparsity caused by the constraint is not complete; that is, not the majority of entries in the adjacency matrix are zero. Given this and coupled with the fact that in correlation networks covariates are correlated and most of them are not zero, methods that can be more robust against over-fragmentation (such as LASSO-based and sparse graphical methods) are not easily applicable.

Here the effect of the topology-constraint in the community structure detection by modularity (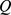) optimization is analyzed, and a strategy to mitigate the over-fragmentation is proposed. Such an effect will be evaluated in a simulation, a protein dataset, and in the 110th US Legislature roll-call votes. In the first two cases, the additional property or constraint property, will be the contact between points in the simulation or residues in the protein. For the roll-call votes, the constraining property is the geographic adjacency of the state of origin of each senator.

## Results and Discussion

**Figure 1:**
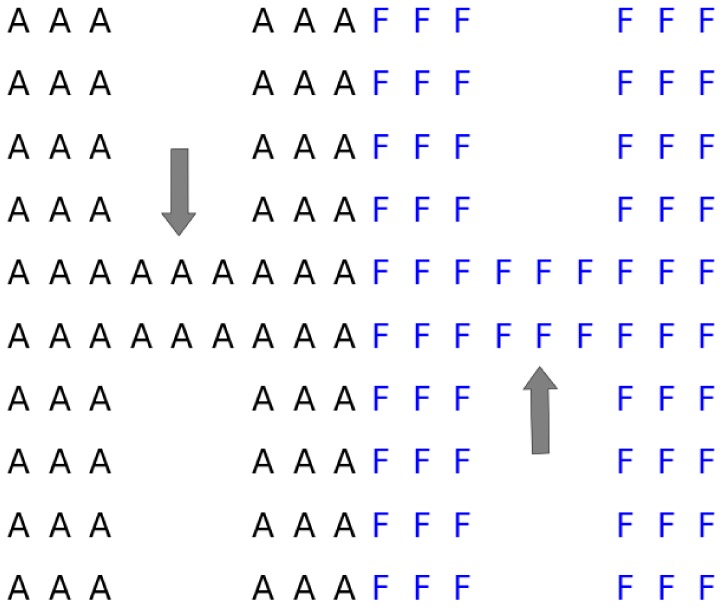
Starting shape for simulation. Letters and colors represent the true clustering. The chokepoints (gray arrows) create weakly linked sub-clusters that should not be fragmented.

To compare with the topology-unconstrained simulations in [1] a shape-structured simulation using Cholesky decomposition (See Methods) is developed. The simulation uses two contiguous letters “H” (Figure 1) to create a heterogeneous shape. The topology constraint is based on contacts since the points in simulation lay on a unit grid. The shape was chosen since it creates a point of contact between the two clusters as well as bottlenecks of contacts which make it a more difficult clustering problem for the topology constraint.

Table 1 shows the results of the performance (mean F-score ± standard deviation; refer to Methods for details) of the methods in [1] in a topology (contacts) constrained simulation. As can be seen, the results here differ from that in [1] simulations, which has no contact constraints. It appears that the reduced number of edges, given the constraint, creates an over-fragmentation by the modularity optimization that cannot be corrected by the 95% confidence permutational t-test reported by [1].

**Table 1:** Performance of the structured simulation without LDA pre-filtering.

		Cluster A								
	Corr.	0.15	0.2	0.25	0.3	0.35	0.4	0.45	0.5	0.55
	0.15	0.80±0.15	0.83±0.12	0.83±0.09	0.79±0.14	0.82±0.13	**0.86**±**0.09**	0.80±0.11	0.81±0.12	0.83±0.12
	0.20	**0.86**±**0.11**	**0.85**±**0.12**	**0.87**±**0.13**	0.83±0.11	**0.85**±**0.12**	0.81±0.09	0.77±0.14	0.78±0.12	0.82±0.11
	0.25	**0.86**±**0.13**	**0.86**±**0.12**	0.83±0.13	0.82±0.14	0.81±0.12	0.74±0.14	0.82±0.13	0.81±0.11	0.82±0.07
	0.30	**0.85**±**0.12**	**0.88**±**0.10**	**0.86**±**0.10**	0.81±0.13	0.83±0.10	**0.85**±**0.13**	0.83±0.11	0.77±0.11	0.81±0.11
	0.35	0.80±0.13	**0.85**±**0.11**	0.84±0.11	0.82±0.14	0.83±0.12	0.81±0.11	0.76±0.12	0.79±0.11	0.77±0.11
	0.40	0.80±0.12	**0.86**±**0.11**	**0.89**±**0.11**	**0.86**±**0.13**	0.81±0.13	0.84±0.09	0.80±0.13	0.76±0.10	0.74±0.10
	0.45	0.83±0.11	**0.85**±**0.08**	**0.88**±**0.10**	0.82±0.13	0.80±0.15	0.84±0.09	0.81±0.14	0.80±0.11	0.73±0.12
	0.50	0.78±0.11	0.81±0.11	0.81±0.14	0.79±0.11	0.82±0.13	0.80±0.11	0.81±0.08	0.80±0.08	0.79±0.09
	0.55	0.79±0.12	0.83±0.12	0.78±0.11	0.84±0.09	0.81±0.13	0.80±0.13	0.79±0.12	0.77±0.13	0.75±0.12
	0.60	0.76±0.12	0.82±0.08	0.80±0.10	**0.86**±**0.07**	0.79±0.10	0.80±0.10	0.78±0.15	0.72±0.13	0.75±0.12
	0.65	0.83±0.08	0.82±0.12	0.79±0.13	0.79±0.11	0.83±0.12	0.79±0.12	0.83±0.10	0.81±0.14	0.78±0.10
	0.70	0.80±0.10	0.79±0.11	0.79±0.11	0.81±0.15	0.79±0.12	0.78±0.13	0.78±0.13	0.83±0.09	0.81±0.13
	0.75	0.79±0.12	0.82±0.10	0.82±0.13	0.76±0.12	0.80±0.11	0.80±0.13	0.77±0.11	0.77±0.11	0.83±0.08
	0.80	0.76±0.14	0.80±0.12	0.80±0.12	0.75±0.11	0.78±0.11	0.73±0.13	0.82±0.13	0.76±0.13	0.77±0.12
	0.85	0.81±0.11	0.83±0.08	0.83±0.12	0.76±0.09	0.79±0.11	0.77±0.13	0.78±0.12	0.74±0.12	0.76±0.12
	0.90	0.77±0.11	0.81±0.12	0.78±0.11	0.83±0.15	0.78±0.11	0.76±0.09	0.76±0.15	0.76±0.09	0.79±0.09
	0.95	0.78±0.09	0.79±0.13	**0.85**±**0.11**	0.80±0.13	0.79±0.13	0.73±0.11	0.70±0.11	0.73±0.12	0.77±0.10
Cluster B		Continuation Cluster A								
	Corr.	0.6	0.65	0.7	0.75	0.8	0.85	0.9	0.95	
	0.15	0.80±0.12	0.81±0.16	0.81±0.12	**0.88**±**0.11**	0.83±0.08	0.83±0.10	0.80±0.09	0.84±0.09	
	0.20	0.80±0.09	0.81±0.10	0.84±0.09	0.82±0.13	0.82±0.09	**0.85**±**0.09**	0.79±0.11	0.77±0.08	
	0.25	0.81±0.12	0.79±0.13	0.83±0.12	0.76±0.10	0.82±0.09	0.79±0.12	0.77±0.12	0.81±0.10	
	0.30	0.81±0.08	0.76±0.12	0.77±0.11	0.82±0.11	0.79±0.12	0.79±0.11	0.80±0.15	0.80±0.10	
	0.35	0.79±0.12	0.76±0.10	0.77±0.12	0.83±0.14	0.76±0.14	0.80±0.11	0.78±0.14	0.82±0.10	
	0.40	0.79±0.13	0.74±0.12	0.79±0.10	0.75±0.12	0.71±0.11	0.75±0.11	0.75±0.11	0.81±0.09	
	0.45	0.79±0.13	0.78±0.13	0.76±0.12	0.72±0.14	0.84±0.11	0.74±0.13	0.79±0.12	0.76±0.11	
	0.50	0.79±0.13	0.80±0.12	0.78±0.10	0.82±0.12	0.72±0.13	0.78±0.12	0.78±0.11	0.77±0.14	
	0.55	0.79±0.13	0.79±0.11	0.78±0.12	0.73±0.13	0.76±0.11	0.77±0.09	0.78±0.11	0.79±0.14	
	0.60	0.77±0.12	0.79±0.10	0.77±0.09	0.74±0.11	0.74±0.10	0.76±0.12	0.73±0.10	0.74±0.13	
	0.65	0.79±0.11	0.78±0.14	0.81±0.11	0.77±0.14	0.74±0.12	0.72±0.11	0.76±0.10	0.69±0.13	
	0.70	0.78±0.10	0.81±0.11	0.81±0.12	0.80±0.11	0.77±0.13	0.78±0.11	0.66±0.11	0.75±0.10	
	0.75	0.81±0.10	0.79±0.11	0.77±0.13	0.80±0.10	0.80±0.14	0.78±0.10	0.69±0.11	0.75±0.14	
	0.80	0.79±0.11	0.77±0.14	0.83±0.08	0.77±0.14	0.79±0.12	0.79±0.11	0.77±0.12	0.80±0.10	
	0.85	0.78±0.12	0.78±0.13	0.82±0.09	0.79±0.13	0.83±0.10	0.81±0.11	0.80±0.10	0.80±0.12	
	0.90	0.83±0.10	0.77±0.15	0.76±0.11	0.81±0.10	0.79±0.09	0.83±0.12	0.78±0.13	0.81±0.08	
	0.95	**0.85**±**0.12**	0.77±0.13	0.75±0.14	0.78±0.10	0.80±0.10	0.84±0.09	0.80±0.10	0.81±0.12	

Mean F-score and standard deviation of 20 replicates of a Cholesky-based structured simulation.

Each entry corresponds to the mean F-Score ± the standard deviation for 20 replicates in each pair of intracorrelations. Corr.  =  Intracorrelation. Bolded numbers correspond to F-Scores higher than 0.85.

### Addressing the over-fragmentation problem: Linear discriminant filtering

Linear discriminants are a standard multivariate statistical tool to reduce the dimensionality by finding a suitable linear subspace in which the the groups or classes are optimally separated by maximizing the variance between groups while minimizing the intraclass variance. It has been commonly used as a preprocessing step in pattern recognition systems [25] and is commonly used in other sciences to explore the variate space to find shared properties of samples and variables [26]. It is based on a linear model where a given dependent variable can be explained by a linear combination of factors given by the independent variables. Such factors can be a clustering scheme itself. By providing a membership vector derived from the optimization of the modularity score, the linear discriminant analysis (LDA) will provide a set of linear discriminants that better fit the data. Such linear discriminants can be analyzed for the differences between groups. When the differences between groups are not large enough given a particular clustering scheme, some collision between classes may occur in which case it can be hypothesized that there is not enough information in the data to support their separation.

After obtaining the membership vector for a topology-constrained dataset, and before performing significance testing as explored in [1], a filtering step is introduced using LDA:

1. Given the membership vector of the modularity optimization, fit the data to the grouping using LDA.
2. Using the first two linear discriminants find the 95% confidence ellipses of each group.
3. Determine if there is a collision between all pairs of ellipses.
4. Merge groups if a collision is found.

The Methods section contains the details for each of these steps. Table 2, shows the results of 20 replicates of the simulation of topology-constrained correlation networks with the implementation of LDA filtering. As can be seen, the improvement is significant (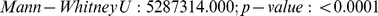) obtaining the true answer in most cases (even in intra-community correlations as low as 0.15). Is important to keep in mind that our simulations also include correlation between clusters (inter-community correlation) drawn from a random uniform distribution with minimum of 0 and maximum of 0.1. This means that the discrimination with the LDA filtering is robust even with correlation noise.

**Table 2:** Performance of the structured simulation using LDA pre-filtering.

		Cluster A								
	Corr.	0.15	0.2	0.25	0.3	0.35	0.4	0.45	0.5	0.55
	0.15	**0.96**±**0.06**	**0.96**±**0.07**	**0.92**±**0.09**	**0.93**±**0.08**	**0.93**±**0.09**	**0.93**±**0.09**	**0.93**±**0.09**	**0.93**±**0.09**	**0.93**±**0.07**
	0.20	**0.96**±**0.06**	**0.97**±**0.07**	**0.97**±**0.06**	**0.98**±**0.05**	**0.96**±**0.06**	**0.94**±**0.11**	**0.95**±**0.09**	**0.96**±**0.06**	**0.97**±**0.06**
	0.25	0.92±0.09	**0.99**±**0.04**	**0.98**±**0.05**	**0.99**±**0.03**	**0.96**±**0.07**	**0.93**±**0.10**	**0.92**±**0.12**	**0.96**±**0.04**	**0.95**±**0.06**
	0.30	**0.94**±**0.07**	**0.97**±**0.05**	**0.93**±**0.13**	**0.95**±**0.10**	**0.93**±**0.07**	**0.91**±**0.11**	**0.96**±**0.07**	**0.93**±**0.10**	**0.96**±**0.08**
	0.35	**0.95**±**0.07**	**0.97**±**0.05**	**0.94**±**0.10**	**0.93**±**0.10**	**0.94**±**0.12**	**0.94**±**0.07**	**0.94**±**0.10**	**0.96**±**0.07**	**0.90**±**0.10**
	0.40	**0.93**±**0.07**	**0.95**±**0.10**	**0.96**±**0.07**	**0.92**±**0.10**	**0.96**±**0.06**	**0.96**±**0.07**	**0.94**±**0.07**	**0.95**±**0.07**	**0.96**±**0.09**
	0.45	**0.94**±**0.09**	**0.96**±**0.08**	**0.93**±**0.09**	**0.95**±**0.09**	**0.93**±**0.09**	**0.92**±**0.14**	**0.93**±**0.10**	**0.96**±**0.11**	**0.92**±**0.10**
	0.50	**0.93**±**0.07**	**0.96**±**0.09**	**0.92**±**0.08**	**0.92**±**0.11**	**0.93**±**0.10**	**0.92**±**0.10**	**0.93**±**0.10**	**0.94**±**0.10**	**0.95**±**0.07**
	0.55	**0.95**±**0.08**	**0.96**±**0.06**	**0.96**±**0.06**	**0.96**±**0.09**	**0.93**±**0.11**	**0.93**±**0.10**	**0.91**±**0.12**	**0.87**±**0.09**	**0.92**±**0.12**
	0.60	**0.93**±**0.08**	**0.93**±**0.09**	**0.91**±**0.10**	**0.96**±**0.06**	**0.95**±**0.06**	**0.94**±**0.09**	**0.96**±**0.07**	**0.96**±**0.07**	**0.93**±**0.12**
	0.65	**0.96**±**0.06**	**0.95**±**0.05**	**0.94**±**0.08**	**0.96**±**0.07**	**0.91**±**0.10**	**0.95**±**0.07**	**0.93**±**0.11**	**0.90**±**0.12**	**0.92**±**0.09**
	0.70	**0.94**±**0.06**	**0.94**±**0.09**	**0.96**±**0.05**	**0.96**±**0.07**	**0.91**±**0.12**	**0.92**±**0.09**	**0.94**±**0.09**	**0.91**±**0.11**	**0.92**±**0.06**
	0.75	**0.91**±**0.08**	**0.97**±**0.05**	**0.96**±**0.07**	**0.94**±**0.07**	**0.94**±**0.06**	**0.92**±**0.08**	**0.91**±**0.08**	**0.94**±**0.08**	**0.95**±**0.06**
	0.80	**0.94**±**0.08**	**0.93**±**0.09**	**0.88**±**0.10**	**0.89**±**0.10**	**0.92**±**0.10**	**0.98**±**0.04**	**0.94**±**0.10**	**0.91**±**0.10**	**0.91**±**0.10**
	0.85	**0.95**±**0.08**	**0.89**±**0.12**	**0.93**±**0.08**	**0.93**±**0.08**	**0.92**±**0.10**	**0.90**±**0.08**	0.87±0.11	**0.95**±**0.07**	**0.93**±**0.08**
	0.90	**0.93**±**0.09**	**0.94**±**0.07**	**0.94**±**0.06**	**0.96**±**0.06**	**0.93**±**0.09**	**0.94**±**0.07**	**0.96**±**0.08**	**0.90**±**0.10**	**0.93**±**0.09**
	0.95	**0.95**±**0.07**	0.92±0.09	**0.95**±**0.07**	**0.90**±**0.07**	**0.93**±**0.08**	**0.92**±**0.08**	**0.93**±**0.07**	**0.94**±**0.08**	**0.94**±**0.07**
Cluster B		Continuation Cluster A								
	Corr.	0.6	0.65	0.7	0.75	0.8	0.85	0.9	0.95	
	0.15	**0.94**±**0.08**	**0.92**±**0.13**	**0.92**±**0.09**	**0.91**±**0.10**	**0.91**±**0.08**	**0.94**±**0.09**	**0.96**±**0.06**	**0.96**±**0.06**	
	0.20	**0.95**±**0.09**	0.92±0.08	**0.91**±**0.08**	**0.94**±**0.06**	**0.91**±**0.09**	**0.97**±**0.05**	**0.95**±**0.08**	**0.92**±**0.09**	
	0.25	**0.93**±**0.07**	**0.94**±**0.08**	0.87±0.11	**0.92**±**0.10**	**0.95**±**0.06**	**0.96**±**0.08**	**0.95**±**0.07**	**0.94**±**0.08**	
	0.30	**0.92**±**0.11**	**0.94**±**0.07**	**0.94**±**0.09**	**0.91**±**0.12**	**0.93**±**0.09**	**0.92**±**0.09**	**0.94**±**0.08**	**0.92**±**0.06**	
	0.35	**0.96**±**0.06**	**0.93**±**0.07**	**0.93**±**0.08**	**0.93**±**0.06**	**0.95**±**0.06**	0.93±0.09	**0.94**±**0.10**	**0.94**±**0.07**	
	0.40	**0.96**±**0.05**	**0.91**±**0.12**	**0.89**±**0.10**	**0.91**±**0.11**	**0.97**±**0.06**	**0.90**±**0.11**	**0.93**±**0.05**	**0.89**±**0.09**	
	0.45	**0.94**±**0.11**	**0.93**±**0.07**	**0.92**±**0.08**	**0.95**±**0.08**	**0.92**±**0.11**	**0.92**±**0.12**	**0.94**±**0.07**	**0.94**±**0.07**	
	0.50	**0.97**±**0.05**	**0.93**±**0.09**	**0.94**±**0.09**	**0.91**±**0.11**	**0.90**±**0.12**	**0.91**±**0.07**	**0.92**±**0.09**	**0.94**±**0.09**	
	0.55	**0.94**±**0.09**	**0.93**±**0.11**	**0.93**±**0.11**	**0.91**±**0.11**	**0.89**±**0.14**	**0.90**±**0.09**	**0.94**±**0.08**	**0.93**±**0.07**	
	0.60	**0.92**±**0.10**	**0.96**±**0.10**	**0.92**±**0.09**	**0.88**±**0.13**	**0.94**±**0.10**	**0.91**±**0.10**	**0.94**±**0.08**	**0.91**±**0.09**	
	0.65	**0.95**±**0.09**	**0.94**±**0.08**	**0.90**±**0.12**	**0.93**±**0.11**	**0.91**±**0.08**	**0.93**±**0.12**	**0.95**±**0.07**	**0.93**±**0.10**	
	0.70	**0.95**±**0.06**	**0.92**±**0.11**	**0.91**±**0.09**	**0.91**±**0.12**	**0.92**±**0.08**	**0.93**±**0.08**	**0.96**±**0.07**	**0.94**±**0.11**	
	0.75	**0.94**±**0.07**	**0.90**±**0.14**	**0.95**±**0.08**	**0.91**±**0.12**	**0.92**±**0.08**	**0.94**±**0.08**	**0.95**±**0.06**	**0.94**±**0.10**	
	0.80	**0.88**±**0.11**	**0.95**±**0.07**	**0.88**±**0.10**	**0.91**±**0.11**	**0.96**±**0.09**	**0.93**±**0.10**	**0.90**±**0.12**	**0.97**±**0.05**	
	0.85	**0.94**±**0.08**	**0.91**±**0.09**	**0.93**±**0.09**	**0.93**±**0.11**	**0.88**±**0.14**	**0.91**±**0.11**	**0.92**±**0.11**	**0.95**±**0.08**	
	0.90	**0.96**±**0.06**	**0.93**±**0.10**	**0.92**±**0.11**	**0.90**±**0.12**	**0.93**±**0.09**	**0.95**±**0.07**	**0.95**±**0.08**	**0.97**±**0.08**	
	0.95	**0.91**±**0.09**	**0.95**±**0.06**	**0.94**±**0.07**	**0.98**±**0.04**	**0.97**±**0.06**	**0.94**±**0.06**	**0.99**±**0.04**	**0.99**±**0.04**	

Mean F-score and standard deviation of 20 replicates of a Cholesky-based structured simulation.

Each entry corresponds to the mean F-Score ± the standard deviation for 20 replicates in each pair of intracorrelations. Corr.  =  Intracorrelation. Bolded numbers correspond to a significant (Mann-Whitney pvalue 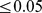) improvement of the F-Score with respect to the one obtained without LDA-prefiltering.

Despite the usefulness of the LDA in topology-constrained correlation network analysis, it is important to state that in a fully or nearly-fully connected graph, LDA tends to cluster everything in a single group. This is particularly true when the variance is small (data not shown). However, as shown in Table 2, LDA dramatically increases the performance when there is a topology constraint in the graph.

### Case studies

Now some case studies that have been analyzed previously [1], [27] will be considered. In this section it will shown how there are some real cases in which a topology-constrained correlation network community structure is over-fragmented. It is also shown how LDA can address fragmentation without systematically merging every partition scheme.

#### Voting in the United States 110th Senate

**Figure 2:**
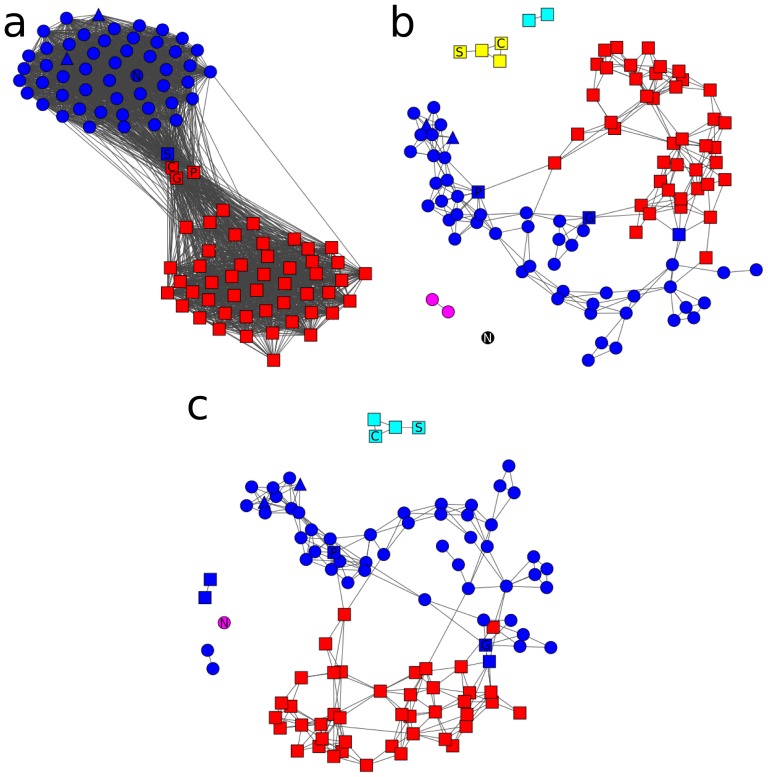
Networks of correlations of roll-call votings in the 110th US senate. 2a: Correlation network without state neighborhood constrain and without the use of LDA pre-filtering; 2b: Correlation network with state neighborhood constrain but without the use of LDA pre-filtering; 2c: Correlation network with state neighborhood constraint and using of LDA pre-filtering. The nodes are colored by cluster and each party is denoted with a given shape. Triangle: Independent; Square: Republican; Circle: Democrat. Letters inside nodes represents some senators names mentioned in text. S: Snowe; N: Nelson (FL); G: Smith (OR); Collins (ME); P: Specter (PA).

**Figure 3:**
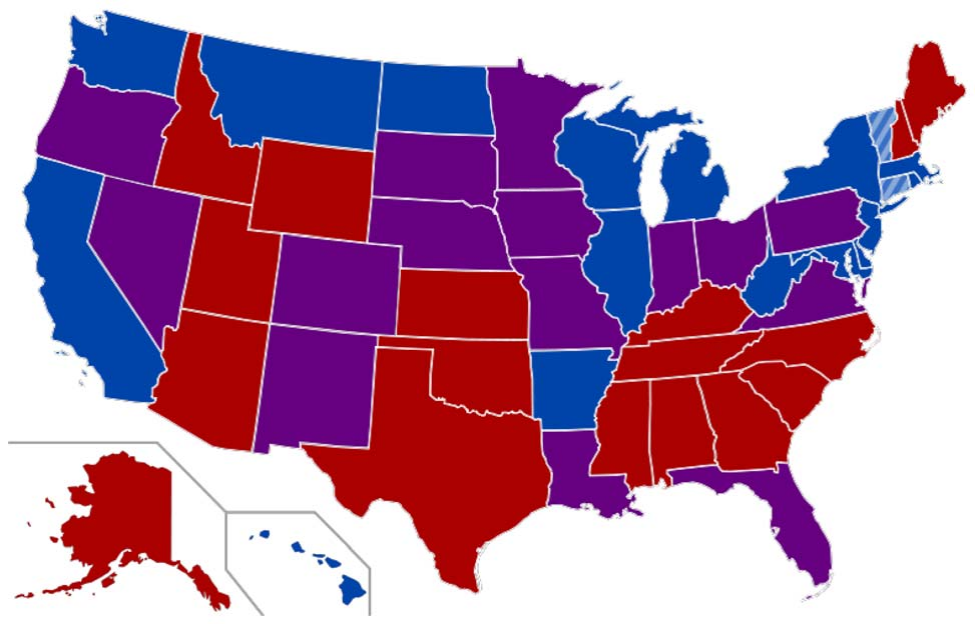
110th US Congress Senate. USA map colored by the party who holds the seats in the 110th Senate (between January 3, 2007, and January 3, 2009). Blue: fully Democratic state; Red: Fully Republican state; Purple: Half Republican, half Democratic; Striped blue: Independent senator. Image taken from http://commons.wikimedia.org/wiki/File:110th_US_Congress_Senate.svg.

A great effort has been placed into analyzing the political partisanship in the US congress, particularly on how polarized Legislatures can influence the voting on non-particular issues [28]. In the 110th Legislature of the United States, in the second government of G.W. Bush, the polarization was evident. It has been suggested that in highly polarized Legislatures the representatives tend to vote more strongly with their party. Figure 2 shows that not only the polarization played an important roll. In Figure 2a, it is evident that the vote of individual representatives fell along party lines. Each color represents the cluster and the party, with the exception of the independent representatives whose votes are indistinguishable from the Democrats, and Senator Snowe, that despite being a Republican voted more similarly to Democrats. Figure 2a). If this correlation graph is constrained to geographical adjacency (i.e. neighboring states), the clustering is modified. In Figure 2b six clusters are found. The singleton (black node) corresponds to senator Nelson, a Democrat representative the Republican dominated region of Florida (South USA; Figure 3). Nodes in cyan and magenta correspond to Alaska and Hawaii, which have no neighbors. The yellow cluster includes Maine and New Hampshire senators who (as can be seen in Figure 3) are Republicans in a Democrat/independent neighborhood. When the LDA prefiltering is used (Figure 2c), the clusters corresponding to Hawaii and Alaska are merged with the blue cluster which mainly contains Democrats, while Alaska had a Republican representation. However in Figure 2a, the Alaskan representatives had a voting profile closer to the Democrat along with Senator Collins (Maine), Senator Specter (Pennsylvania) and Senator Smith (Oregon), who were also Republicans with an intermediate voting profile between Democrats and their party. In figure 2c, Senator Collins (Maine) and Senator Specter (Pennsylvania) actually cluster with a few other Republicans and Democrats following a neighborhood voting profile. Despite polarization, there is still a neighborhood signal driving some of the votings. However, most Republicans and Democrats have a clear partisan profile of voting, and the differences rely on particular bills and motions that might have a regional scope.

In this example it can be seen that after the LDA filtering, the number of clusters obtained is reduced. Given the results of the simulation show that the heuristic to optimize 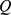 does overfragment the graph, the observed reduction is likely a more accurate description of the community structure giving a regional focus. The LDA filtering proposed here have no information of the topology constraint, therefore the results shown in this section demonstrate that there is a geographic signal in the US votes, and that does not follow a party-strict pattern. In this particular case, the correlation graph in Figure 2a shows that the polarization plays the major role, splitting most Democrats and Republicans in different groups. However, Figures 2b and 2c show that a regional bias remains in some of the motions voted.

#### 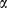-Amylase homologs sub-domain architecture

**Figure 4:**
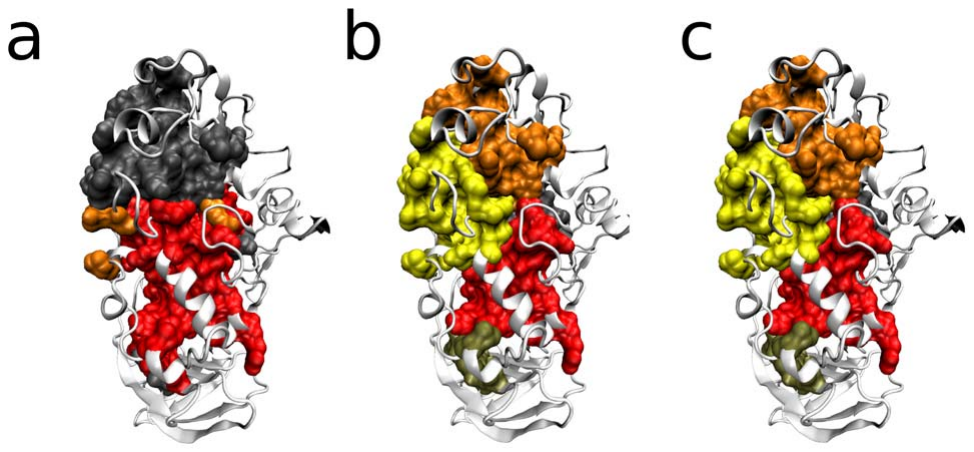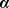-amylase homologs. Clusters (modules) found in an extension of the modularity inference performed in [1], inclusing 135 homologs of the catalytic domain of the 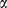-amylase. a) Modules inferred without constraining the topology with inter-residue contacts. b) Modules inferred constraining the topology in A with inter-residue contacts. c) Modules inferred by prefiltering the results in B, before significance testing.

In Hleap et al. [1], a dataset of 85 protein structures was analyzed to find a sub-domain architecture. They found four significant clusters, one of which comprises the minimum functional TIM-barrel [1]. In this manuscript that search has been broaden gathering 135 structures. To show a biological application of the LDA prefiltering, the algorithm described in [1] without contacts restrains was performed, with inter-residue contacts constraint, and the latter with LDA pre-filtering. Figure 4 shows the results for this case, where each color represents a cluster of residues within the protein. In the absence of contact restrains (Figure 4a) bigger clusters are found. Some clusters are made of disconnected components (orange cluster). There are significant smaller clusters than in the other cases (Figures 4b and 4c), and the biological meaning for the lack of contiguity is obscure. It can be ascribed that disjoint components in a cluster reflect a higher level community, which is not interesting from a protein modularity perspective. Figure 4b, shows the result for the same algorithm, when considering topology constraint based on the inter-residue contacts. Here, more sensible results are gathered returning the minimal functional TIM barrel topology obtained in [1] (yellow cluster). Figure 4c corresponds to the same topology-constrained network in Figure 4b, but with LDA pre-filtering, however the result is identical. This suggests that the LDA-filtered community structure at the protein level is strong and significant enough to avoid merging. This observation makes sense since Hleap et al. [1] were testing for correlation among residues and this information can be correlated with the contact between them. It is also important to state that when no over-fragmentation occurs (like in this particular dataset) LDA will not affect the result.

## Conclusions

Here, by means of structured simulations, it is shown that topological constraints in a correlation network can lead to over-fragmentation, which supports the claims in [24].

It also has been shown that topological constraints can be used to mine correlation graphs to obtain particular insights. The Roll-Call voting results demonstrate that there is a more complex structure than partisan politics alone, and in the LDA-filtered graph there is less fragmentation than in the non-filtered one. The inter-residue correlation network in protein structures needs to be considered with contacts to obtain biologically meaningful results. This can be a problem if artificial fragmentation is being created. However, it has been shown that LDA filtering does not merge clusters that were found to be meaningful in the first place.

It can be argued that other methods, such as sparse graphical models and LASSO-based methods [15], [29], exist to cope with the over-fragmentation in sparser graphs. However, correlation graphs normally do not fulfill the assumptions of such methods like independence of the variables, *a priori* knowledge of some community properties, and a high degree of sparceness of the covariation among variables. Furthermore, optimization of 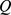 has been an important tool for community detection in graph theory. Solving the problem of over-fragmentation by LDA and statistical testing is an important contribution to the study of correlation graphs in a data-driven way, without the need of a model, and where the distributional properties of the variables are not the main driving force of inference.

## Methods

### Multivariate normal structured simulations

To create the true clustering shown in Figure 1, the same approach done in [1] non-structured or topology-unconstrained simulations will be applied. However, to retain the shape (topology), the following procedure will be done:

1. Create a 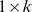 vector with original shape coordinates (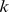).
2. Create a 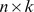 shape matrix, where each row is a repetition of the vector in the previous step. 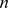 is the number of desired samples.
3. Obtain a 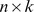 multivariate normal (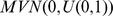) matrix as performed in [1].
4. Create a 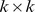 correlation matrix following the structure of each true module.
5. Perform the Cholesky decomposition on the random matrix (multivariate normal matrix) as explained in [1].
6. Sum the factorized random (and therefore now correlated) and shape matrices.

For the Cholesky decomposition, the intracorrelation in both clusters was controlled, starting in 0.15 to 0.95, in 0.05 increments. The intercorrelations in between clusters were drawn from a uniform distribution (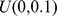). Given that [1] showed that 500 samples were enough to resolve most of the correlations, only as many samples were used.

This simulation was repeated 20 times for each intracorrelation pairs.

### Performance measure

To quantify the performance of the simulation, an F-Score was calculated as: 
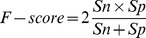
(2)where 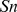 stands for sensitivity which can be expressed as 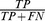; and 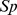 stands for specificity which can be estimated as 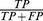.

In all cases, 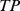 are the true positives, 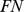 are the false negatives, and 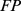 are the false positives.

The results of the 20 simulations are summarized as the mean F-score ± the F-score standard deviation for each intracorrelation pair.

### Contact definition

In structured (shape-defined) datasets, a contact matrix can be inferred. Each point in a given configuration is said to be in contact with any other point in the dataset if the distance between a given pair is not greater than one unit plus the standard deviation of the simulation. This holds true only if the shape being constructed lays on a grid of one unit per square cell (like ours does). In the Roll-Call voting dataset, the contact was defined as touching (neighbors) states. In the case of the protein dataset, the contact matrix was inferred as in Hleap et al. [1].

### Filtering the 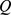 optimization output

The output of the modularity (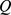) optimization developed by [8] is a membership vector. Here as in [1], the optimization is performed using a fast-greedy algorithm, which has been shown to be a good and fast heuristic for the optimization of 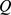 [30]. After such a membership vector is obtained, the refinement proposed by [1] can be performed. However, some over-fragmentation may occur when a topology-constrained graph is used. To deal with this issue, here it is proposed a Linear Discriminants (LD) pre-filtering of the modularity membership vector.

#### Linear Discriminant Analysis (LDA)

The LDA for the present paper was performed using the lda function available in the package MASS [31] in R [32]. Here the fit will be done between the correlation magnitude matrix (as performed in [1]), where each entry row/column corresponds to each variable, and each entry is the magnitude of the correlation vector as the square root of the sum of squared correlations in each dimension (X, Y for 2D, and X, Y, Z for 3D). The latter two cases are generalizations of the simpler case of one dimension in which case the data is the 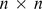 correlation matrix, 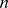 being the variables in the dataset. In any of the cases, a fisher transformation and a significant test of the correlation is performed, as suggested in [1]. This data matrix is the same matrix that represents the graph, where the non-zero entries correspond to an edge and the actual value represents the weight of that edge.

#### Collision test and membership refinement

After the first two LD are obtained, a 95% confidence ellipse is computed. Here, the package ellipse [33] implemented in R [32] is used to compute the ellipses. After the ellipse have been estimated, a collision test is made. A point will be inside or at the edge of any given ellipse if the following inequality [34] is satisfied: 
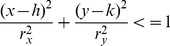
(3)where 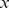 and 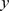 are the coordinates of a given point, 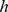 and 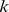 are the coordinates of the center of the ellipse, and 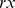 and 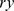 are the semi-minor and semi-major axes of the ellipse.

If the inequality in equation 3 is satisfied, the two ellipses are colliding and therefore the groups/classes they represent should be merged, otherwise the groups are not touched.

With this approach some of the over-fragmentation created by the lost of edges in a topology-constrained network might be dealt with.

### Case studies datasets

#### Voting in the United States 110th Senate

The Roll-Call voting of 110th United States Senate (available online at [35] or in Supporting Information File S1) was used to construct the network. First a data matrix is created where each row represents each senator and each column represents a vote for a given motion or amendment. With that data matrix a correlation matrix 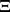 is created, where each entry have been tested for significance using a Z test of a fisher transformation of the correlation. If the significance test failed, the corresponding entry is set to zero, otherwise the correlation value is recorded. Let 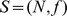 be an undirected graph, where 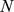 is a list of nodes (senator) and 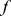 is a function 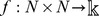 that assigns an edge weight to each senator pair. An edge 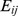 is assigned only if 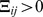. To create a topology-constrained graph a fixed topology accounting for neighboring states is applied to the edge assignment as an extra condition. In the topology-constrained weighted network, an edge will be drawn only if 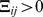, *and if* the senators represent neighboring states. This constraint will allow to test the hypothesis if there is any subdivision that is determined by the geography more than by only party affiliation.

#### 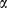-Amylase structures homologs

The 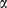-Amylase-like family catalyzes the hydrolysis of 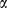-(1,4) glycosidic bonds of polysaccharides, therefore being classified as glycoside hydrolases [36] in the family 13 [37]. It is a multi-reaction catalytic family since its members can catalyze different reactions (hydrolysis, transglycosylation, condensation and cyclization) [38]. All members of this family share a symmetrical TIM-barrel (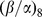) catalytic domain [39], including those without any catalytic activity [40]. This fold is highly versatile and widespread among the structurally characterized enzymes, being present in almost 10% of them [41]–[44]. There has been a debate about the type of evolution that this fold has been through: convergent, divergent or a mixture of both mechanisms [41]. However, there is some evidence suggesting the divergent evolution hypothesis is the most likely [42]. The catalytic activity and substrate binding residues occurs at the C-termini of 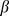-strands and in loops that extend from these strands [39]. The catalytic site includes aspartate as a catalytic nucleophile, glutamate as an acid/base, and a second aspartate for stabilization of the transition state [45]. The catalytic triad plus an arginine residue are totally conserved in this family across all catalysis-active members [37].

In [1], the protein structures belonging to the 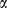-Amylase catalytic domain were gathered from the Homstrad database [46] and these seeded a Blast search restricted to the protein data bank. Here, the search is broaden by seeding a PSI-BLAST [47] search with a PFAM [48] seed alignment of 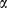-Amylase structures (PFAM code PF00128). The PSI-BLAST search was restricted to structures available at the protein data bank (http://www.rcsb.org/pdb/). There were in total 135 structures gathered which homology and membership to the 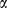-amylase family (the Glycoside Hydrolase Family 13, GH13) was guaranteed (Available in File S1).

Those 135 structures were aligned using the algorithm proposed by [49] that modifies the pairwise MATT flexible structure aligner [50] to complete the multiple structure alignment.

After the alignment, the procedure explained in [1] was used, where the coordinates of the centroid of homologous residues are recorded in a data matrix. The graph construction is performed as before, but one correlation matrix is created per dimension, and then the matrix of magnitudes of the correlation vectors (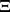) is computed as the euclidean distance between the three matrices. Edges will be assigned, as before, if two residues correlate *and if* they are in contact in the structure (topology constraint).

## Supporting Information

File S1 **Data File.** The data is available as supporting information as a compressed TAR file named File S1.tar.gz containing the files Amy135.gm and sen110kh.2008.USA.roll.call.txt. **File sen110kh.2008.USA.roll.call.txt.** It contains the information of the Roll-Call votings in 2008 for the US Senate. This information is available also in VoteView [35]. The file is space-delimited text file where each line represents a Senator. The first field corresponds to the Senator's code, followed by the state they represent. After the state, a number indicating party affiliation, followed by the lastname of the Senator. The last field correspond to the Roll-Call votes. **File Amy135.gm.** It contains the centroid coordinates in a semicolon-delimited format. In this format the first field correspond to the name of the structure and the X, Y, and Z coordinates for the centroid of each homologous aminoacids are stored sequentially. There is one line per structure (135 in this dataset), and 3 times the number of homologous residues coordinates entries.
(GZ) Click here for additional data file.
